# How and When Does Inclusive Leadership Curb Psychological Distress During a Crisis? Evidence From the COVID-19 Outbreak

**DOI:** 10.3389/fpsyg.2020.01898

**Published:** 2020-08-06

**Authors:** Fawad Ahmed, Fuqiang Zhao, Naveed Ahmad Faraz

**Affiliations:** School of Management, Wuhan University of Technology, Wuhan, China

**Keywords:** inclusive leadership, work engagement, psychological distress, self-sacrifice, the COVID-19, social exchange theory, mental health, health care workers

## Abstract

Traumatic events such as a pandemic shatter the assumption of the workplace as a safe place. Nurses face risks of life-threatening infection, which can create psychological distress. Quality of care for infected patients depends on mental well-being of nurses which calls for research on predictors of stress among health care workers. Responding to a call for research on the effects of leadership styles on psychological distress during traumatic events, this paper uses the theoretical lens of social exchange theory and contributes to literature on relationships between inclusive leadership, psychological distress, work engagement, and self-sacrifice. Participants of this cross sectional study included 497 registered nurses from five hospitals in Wuhan. Data were collected with temporal separation through an online questionnaire. Partial least-squares structural equation modeling was used to analyze data. Results show inclusive leadership has a significant negative relationship with psychological distress. Work engagement mediates this relationship, and nurses’ self-sacrificial behavior moderates it. Findings indicate inclusive leadership style serves as a sustainable mechanism to reduce psychological distress during pandemics. It can operationalize the delivery of mental health support in real-time in work settings. Results provide empirical support for social exchange theory through high work engagement to help control psychological distress among nurses.

## Introduction

Chinese nurses have been exposed to life-threatening occupational risks during the COVID-19 pandemic since it was first reported in December 2019 in Wuhan City, P.R. China. Such traumatic events can shatter their view of the world as a safe place (Janoff-Bulman, 1992). There were five new cases reported in Wuhan during the week of May 2020, 1 month after the lockdown restrictions were lifted (Xinhua, 2020); bringing the overall confirmed infections to 88,423 and 4,634 reported deaths (China Daily, 2020). The COVID-19 has spread beyond China as a global pandemic across continents. As of May 20, 2020, the United States of America (United States) reported 1,535,336 infected cases, Russia 290,678, Spain 278,188, United Kingdom 246,406, Italy 225, 886, Germany 177,213, France 142,903 and Iran 122,492, to name a few countries. The USA and the UK have the highest death tolls with 90,578 and 34,796 reported deaths, respectively (World Health Organization [WHO], 2020). More than 3,300 health care workers in China (Moazzami et al., 2020) and at least 90,000 worldwide (Mantovani, 2020) have been infected while treating patients during this outbreak. A recent multinational study found that 5.3% of health care workers screened positive for moderate to very severe depression, 8.7% for moderate to extremely severe anxiety and 2.2% for moderate to extremely severe stress during the COVID-19 pandemic (Chew et al., 2020).

Nurses are the front-line workers during emergencies, and the public views them as the most respected, ethical and trustworthy professionals in the health sector (Daly et al., 2020). Nurses in Wuhan have faced loneliness while staying isolated and contained due to the highly contagious nature of the virus (Mo et al., 2020). Some even refrained from food intake to avoid toilet breaks as any breaks required changing protective gear, and some shaved their heads to reduce irritation from sweating (Smith et al., 2020). Psychological distress is a possible risk for nurses because epidemiological work links the onset of depression to exposure to different stressors (Stuke and Bermpohl, 2016). Nurses are involved in physical as well as emotional work, along with social encounters, during routine job tasks (Eriksen et al., 2006). The threat to safety at the workplace brings psychological distress for nurses as they face both psychosocial and mechanical stress at work (Wall et al., 1997).

A recent report on the task-force initiative at the Mount Sinai Health Care System (MSHC) during the COVID-19 pandemic in New York City (Ripp et al., 2020) outlines three priority areas to ensure the emotional well-being of the health care work force including (a) fulfilling basic daily needs; (b) providing enhanced communication for delivery of up-to-date, reliable, and reassuring messages; and (c) developing strong measures for psychosocial and mental health support. This is important because the mental health of nurses is essential to caring for patients of infectious disease. Carryer (2020) argues that nurses frequently do not get to participate in leadership decisions about issues that directly affect them and nursing practices; and the COVID-19 pandemic has brought to light this stark reality.

Leadership roles should be studied because leaders can significantly influence employees’ psychological distress levels (Nielsen et al., 2018). Researchers suggest studies on informal leadership roles (Hannah et al., 2014) along new lines of thinking in nursing leadership (Hutchinson and Jackson, 2013) to explore the multilateral influence mechanism of novel leadership styles in multiple contexts of health care. Wong (2015) calls for more research to develop and test theories of leadership for personnel in informal roles. This study focuses on a relational leadership style called inclusive leadership. The influence of positive leadership styles on employee behavior has been studied in the health sector (Asif et al., 2019; Moon et al., 2019; Yang et al., 2019). However, the development of leadership skills among nurses is inadequate (Fischer, 2017), and there is a scarcity of studies on inclusive leadership style (Zhang et al., 2016). Specifically, leadership’s impact on employees’ mental health and well-being during a traumatic event, disaster, or public health emergency requires further research (Birkeland et al., 2016). Nurses face higher psychological job demands due to occupational hazards during infection control, isolation, and containment.

The relationship between work engagement and job demands has been found to affect psychological distress (Oshio et al., 2018). Adverse patient outcomes are associated with nurses’ depression and anxiety. Work engagement has proven to mediate nurses’ professional practices environment and patient safety (Cheng et al., 2020). Inclusive leaders pay attention to their followers’ needs and are always available to support them (Hollander, 2009), which in turn creates dedication among subordinates. The improvement in vigor, dedication to and absorption in their work resulting from inclusive leadership (Choi et al., 2015) plays an important role in the mental condition of nurses. A more engaged workforce at hospitals decreases the probability of adverse events and enhances patient care (Fischer, 2017). Therefore, it is vital to identify work-related predictors of depressive disorders to devise preventive mechanisms.

Nurses in China, especially those who were at the epicenter, Wuhan, sacrificed their Spring Festival holiday, wedding ceremonies, and family celebrations in the greater interest of the nation. Self-sacrifice has been studied in its broader form from a patriotic and social angle (duPreez, 1996; Belanger et al., 2014) or from a mere leadership perspective (Choi and Mai-Dalton, 1998; Mostafa and Bottomley, 2020). Less is known about the self-sacrificial behavior of nursing and healthcare staff. Nurses are directly involved with patients and prone to infection with this deadly disease.

A majority of existing studies on anxiety, depression, and the psychological impact of the COVID-19 focus on the general public and measure the individual psychological distress level and its comparison across groups based on age, gender, tenure, relationship status, working hours, and socio-demographic elements (Huang and Zhao, 2020; Lee and You, 2020; Mazza et al., 2020; Smith et al., 2020). Although some have studied psychological impacts among health care workers (Kang et al., 2020; Mo et al., 2020), these do not address the predictors and mechanisms that can influence psychological distress among nurses. Currently, there is no known study on the inclusive leadership style and its role in implementing real-time measures of support for mental health among nurses during a crisis. As against most existing studies on leadership that reflect on psychological behaviors after the occurrence of traumatic events, this study was carried out during a public health emergency event in response to calls for research by Birkeland et al. (2016). This study focuses on the psychological distress of nurses, specifically assessed during the COVID-19 pandemic.

The novelty of this study is highlighted by three aspects: first, examination of the effect of nursing leaders’ inclusive behavior on psychological distress; second, description of the mediating role of improved work engagement between inclusive leadership and distress; and third, the illustration of the moderating role of the self-sacrificial behavior of nursing staff. Together, these aspects serve as a mechanism which is helpful in curbing psychological distress during the outbreak. The mental health support guidelines for health care workers devised by the Chinese National Health Commission (NHC, 2020) during the COVID-19 outbreak can be translated into a practical and sustainable delivery method through inclusive leadership style.

## Literature Review and Hypotheses

### Inclusive Leadership and Psychological Distress

Inclusive leadership is defined as words and deeds by a leader indicating an “invitation and appreciation for others’ contributions” (Nembhard and Edmondson, 2006). Inclusive leadership refers to “leaders who exhibit visibility, accessibility, and availability” while interacting with subordinates (Carmeli et al., 2010). Inclusive leadership focuses on practices that value employee diversity in decision-making processes. This makes employees comfortable with sharing opinions without being afraid of power distance or status differences (Hassan and Jiang, 2019). Available literature shows that its relationship has been studied in the past with psychological safety (Mikyoung and Moon, 2019), innovative work behavior (Javed et al., 2019), development of employee belongingness (Randel et al., 2018), change of management (Bowers et al., 2012), and the improvement of subordinates’ creativity (Zhu et al., 2020).

Psychological distress refers to general symptoms of depression and anxiety (Ormel and Schaufeli, 1991). It reflects a stable trait component and a state component, which are both susceptible to change after an external event. Work-related stress may potentially activate dysfunctional intermediary psychological and physiological processes, which can lead to an adverse impact on health of employees (Nielsen et al., 2012).

The social exchange theory (Blau, 1964) puts forth the reciprocity concept, which is applicable to formal relationships at the workplace. Employees feel the need to reciprocate leadership support by behaving positively at the workplace. Research has proven the relationship of supervisory behavior with positive mood outcomes among employees (George and Zhou, 2007). Social support received by nurses from their supervisors is a significant predictor of nurses’ well-being, and support from close colleagues is negatively associated with psychological distress (Van der Heijden et al., 2017). It is safe to assume that this need for support intensifies during emergency situations, such as the COVID-19 pandemic. The relationship of leaders with their subordinates develops and changes over time (Shamir, 2011) – more so during testing periods such as epidemics because nurses experience feelings of vulnerability. The behavior of leaders either exacerbates or ameliorates the consequences of extreme situations and stressful events (Hannah et al., 2009).

The theory of shattered assumptions (Janoff-Bulman, 1992) postulates that “exposure to a traumatic event will shatter the target’s basic cognitive schemas about the world, other people, and ourselves” (p. 53). The COVID-19 outbreak has changed the cognitive schemas and employee perceptions of the world as a safe place. A stable mechanism in the conceptual systems is needed; the absence of such a mechanism results in trauma and health problems (Birkeland et al., 2016). Leadership behaviors can impact the health of employees who experience such traumatic events. Inclusive leadership displays interactional justice and trustworthiness (George and Zhou, 2007), which are helpful in rebuilding the confidence of employees. Inclusive leadership contributes to building the psychological meaningfulness and vitality of employees (Binyamin and Brender-Ilan, 2018) to view the world as a safe place, thereby diminishing the negative psychological impact of the pandemic.

A study in the aftermath of a terrorist attack in Oslo found an association of a higher level of supportive leadership with a lower level of psychological distress (PD) (Birkeland et al., 2015). Prior studies are generally limited to broad daily routines where employees have rare chances of suffering from high PD (Ybema and van den Bos, 2010; Elovainio et al., 2013). On the contrary, nurses experiencing the epidemic in Wuhan are more prone to PD during an extreme situation due to higher job demands with diminishing resources (Yu and Li, 2020) during interactions with patients.

From a psychological point of view, epidemic work demands more mental exertion as compared to the routine. Inclusive leadership (IL) plays a role in curbing such psychological pressures by providing ease and peace of mind to the employees because they believe there is someone whom they can turn to in times of need. Inclusive leaders are inherently keen listeners; thus, most situations of hypertension or mental stress can be alleviated through the regular interaction of subordinates with such leaders. The supportive behavior of inclusive leaders minimizes uncertainty, anxiety, and role stress. Therefore, this study hypothesizes that:

Hypothesis 1: Inclusive leadership (IL) has a negative effect on Psychological Distress (PD).

### Mediating Role of Work Engagement

Research in occupational health has shifted from a disease model toward positive mental health outcomes (Schaufeli, 2004). Work engagement is one such outcome that refers to a state of mind with a sense of fulfillment at the workplace. It has three dimensions, i.e., vigor, dedication, and absorption (Schaufeli and Bakker, 2003). When employees receive support from their organization, leaders, and environmental factors, they tend to show improved engagement in work through a higher level of vigor, a more dedicated approach toward their duties and an increased absorption in their tasks (Bakker and Leiter, 2010).

An occupational cohort study by Oshio et al. (2018) found that work engagement moderates the relationship of psychological distress with workload and time pressure among Japanese respondents. Pepe et al. (2019) observed that work engagement helps reduce psychological distress and improve job satisfaction. The relationship between leadership and work engagement has also been studied in the literature. Another study found that leadership styles predict work attitudes (Shkoler and Tziner, 2020).

Work engagement was also observed to mediate the relationship between transformational leadership and extra role behaviors of nurses (Salanova et al., 2011). IL has been found to moderate the affect of work engagement on positive behavior within workplace (Wang et al., 2019). Work engagement proved to mediate the relationship between humble leadership and innovative behavior (Yang et al., 2019). More engaged health care workers display higher work efficiency and better patient care. Therefore, further research is needed to explore the predictors and outcomes of work engagement in health care.

The social exchange theory (Blau, 1964) posits that it is an employee’s felt obligation to repay an organization if he or she is receiving a high level of support from management (Rhoades and Eisenberger, 2002). Employees tend to develop a level of belief with respect to how much their contribution is valued, as postulated by the organizational support theory (Eisenberger et al., 1986). IL and work engagement are closely knit within the social exchange theory. Inclusive leadership increases the expectation of aid when needed and strengthens employee vigor and self-efficacy (Kurtessis et al., 2017). The theory states that each of the involved parties has a certain set of perceptions with respect to the behavior of the other. Contrary to employees’ expectations, if there is a lack of leader support, there may be a decline in work engagement and job outcomes (Stinglhamber and Vandenberghe, 2003).

Nursing personnel in informal leadership positions must pay attention to their leadership roles and expectations (Wang et al., 2019). This adds meaning to work and reminds nurses about the nature of work and the potential of things to go wrong (Edmondson, 2017). This study makes a contribution in the context of leadership, work engagement, and its impact on psychological distress during epidemics. It helps understand the mechanism that links an inclusive leader’s influence on psychological distress by fostering employees’ occupational well-being. Bergin and Jimmieson (2020) found a negative relationship of psychological strain with stress-related behavior of employees who have experienced a high level of praise and recognition by their supervisors.

An inclusive leader connects with subordinates on an emotional level (Hirak et al., 2012), recognizing subordinates and motivating them toward a positive mindset and behaviors (Javed et al., 2017; Hassan and Jiang, 2019; Mikyoung and Moon, 2019). Their support and attention to followers’ needs help bring about positive moods among employees (Hollander, 2009), which, as a pleasant affective state, allows for thriving within the workplace (Choi et al., 2015). This leads to higher levels of vigor among nurses, who display intense dedication to their duties and become more absorbed in their work, otherwise conceptualized as work engagement (WE). It is a continuing state of mind in which subordinates drive their energy into physical and emotional labor. Therefore, Salanova and Schaufeli (2008) argue that work engagement works as a constant “affective-emotional state without a specific focus” (p. 118) and plays a key role in eliminating the negative effects of more specific states such as psychological distress.

The focus on work and a sense of purpose through improved work engagement positively affects the psychological condition. It can be argued along the same lines that work engagement mediates between inclusive leadership and psychological distress to reduce psychological distress among employees during the epidemic through increased vigor, dedication, and absorption in work. Thus, work engagement is a connecting mechanism between inclusive leadership and positive mindset with lower levels of psychological distress. The above discussion leads us to hypothesize that:

Hypothesis 2: Work Engagement (WE) mediates the relationship between inclusive leadership (IL) and Psychological Distress (PD).

### The Moderating Role of Co-worker Self-Sacrificial Behavior

Belanger et al. (2014) defines self-sacrifice as “the psychological readiness to suffer for a cause” (p. 496). It includes a motivational aspect of readiness, involves a cost such as suffering, and is related to a cause. It has also been termed as a personality trait; whereas, in organizational settings, it refers to abandonment and/or postponement of one’s personal interest, privileges, or welfare (Choi and Mai-Dalton, 1998).

Research has shown that support received from coworkers has an inverse relationship with psychological distress (Van der Heijden et al., 2017). During the COVID-19 outbreak, more than 20,000 Chinese health care workers sacrificed their biggest annual family holiday of the Spring Festival to volunteer for support in the epidemic work in Hubei Province (Huang and Zhao, 2020). This behavior of self-sacrifice and unselfishness is also conceptualized in literature as altruism, which stems from the idea of sacrificing for the nation (duPreez, 1996). Self-sacrifice by colleagues generates an others-centered approach of empathy and has been termed very similar to altruism (Belanger et al., 2014). It manifests a devotion to collective needs and creates a collective identity among nurses, who display group-oriented and collaborative behavior.

Social exchange theory not only posits reciprocity between leadership support and employee behavior, but also applies to peer-support among employees with respect to the principle of moral indebtedness (Mostafa and Bottomley, 2020). Employees take inspiration and give back to the organization through job tasks as well as interpersonal relationships within the workplace, and this give and take is not merely between management and employees but also from employees to other colleagues (Ancarani et al., 2017). Nurses may place particularly high importance on their coworkers’ perceptions of them. This makes it particularly important to ensure that nurses feel supported by their immediate colleagues. These feelings are reciprocal as support received and support provided facilitate feelings of self-sacrifice among nurses who endure the hardship of epidemic work for the sake of others.

**FIGURE 1:**
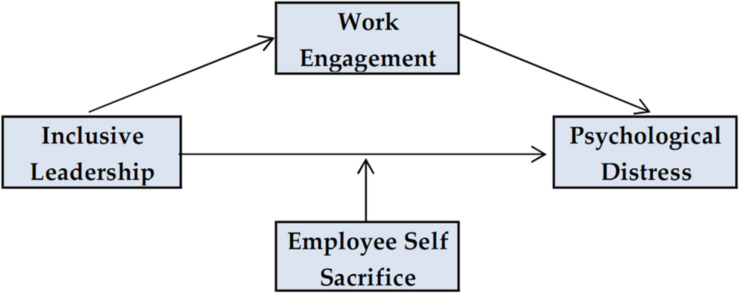
Research Model.

As Brooks et al. (2020) observe, health care workers may show concerns that their workplaces could be understaffed if they were not able to continue work due to stress and that they could cause a higher workload for their colleagues. Thus, nurses feel less worried when they observe self-sacrificial and altruistic behavior around them because it creates positive emotional states and a supportive environment. Based on the above discussion, we hypothesize that nurses’ self-sacrificial behavior will moderate the relationship between inclusive leadership and psychological distress so that the higher level of self-sacrifice will strengthen the negative impact of inclusive leadership on psychological distress leading to reduced levels of distress. Figure 1 presents the research model.

Hypothesis 3: Employee Self-Sacrifice (ESS) moderates the relationship between inclusive leadership (IL) and Psychological Distress (PD) so that the higher level of ESS increases the negative effect of IL on PD while a lower level of ESS decreases the negative effect of IL on PD.

## Materials and Methods

### Sampling Procedure and Participants

Meetings were held with nursing staff chiefs of five hospitals in Wuhan from January 18 to January 20, 2020. Researchers explained the purpose of the research in order to obtain permission. Due to physical mobility restrictions and a subsequent lockdown, a web-based questionnaire was used. One of the authors created a group chat on Wechat, the most commonly used application in China. This group’s Quick Response (QR) code was printed on paper along with a disclosure statement about the purpose of the research, its academic nature, and voluntary participation. Head nurses at the five hospitals placed this QR code and disclosure statement on the notice boards near reporting desks and duty stations of nurses to recruit respondents. Nurses joined the group chat for participation in the study with informed consent. A Mixed/Snowball sampling technique was used, and the researchers asked the participants to invite colleagues other than members of the group chat. The authors explained the procedure to nurses via Wechat and answered their questions before initiating data collection. The timeline for data collection was also provided to participants beforehand. Data were collected in three stages: data for marker variable and demographics were collected at time 1 on February 15, 2020; for inclusive leadership and work engagement at time 2 on February 18; and for psychological distress and self-sacrifice at time 3 on February 21, 2020. At each time, a questionnaire was created through Wenjuan^1^ and the URL was shared via the group chat. Only Wechat identifiers (IDs) of nurses were obtained for use as unique identifiers to match the responses to participants at all time points. During all stages of data collection, one of the authors remained available to answer any queries raised by participants and obtained feedback to remove any possible ambiguities.

A total of 543 responses were received at time 1; 537 responses were matched at time 2 with responses from time 1. Twenty-three responses were unusable and were discarded, yielding 514 responses. These respondents were retained and others were removed from the data collection process. At time 3, the 514 respondents were asked to fill the questionnaire related to inclusive leadership and work engagement. Eventually, 497 responses were deemed usable after screening for unreasonably short duration of responses and missing values (sample profile in Table 1).

**TABLE 1:** Sample profile.

	*n*	%age		*n*	%age
**Gender**			**Education**		
Male	105	21.13%	High school/College	283	56.94%
Female	392	78.87%	Bachelor’s or Above	214	43.06%
**Age (years)**			**Working hours per week**		
≤35	294	59.15%	≤40	308	61.97%
>35	203	40.85%	>40	189	38.03%
**Experience (years)**			**Designations/Cadres**		
<05	260	52.31%	Nurse	258	51.91%
≥5	237	47.69%	Senior nurse	143	28.77%
			Supervisor nurse	96	19.32%

Hospital nurses in China have five career levels: nurse, senior nurse, supervisor nurse, co-chief superintendent nurse, and chief superintendent nurse. Co-chief superintendent and supervisor nurses are usually head nurses (Wang et al., 2019). The data were collected from nurses, senior nurses, and supervisor nurses about the leadership behavior of their superiors, self-sacrificial behavior of coworkers and their own work engagement and psychological distress. Power analysis (Hair et al., 2013) was used by considering the variable with the largest number of predictors. The sample size was well above the threshold of 52 for two predictors for a 0.25 R-squared value at the 0.05 significance level and 80 percent power.

### Measures

*Inclusive Leadership* was measured through a nine-item scale adopted from Carmeli et al. (2010), who used a one-factor model (Cronbach’s alpha = 0.94) on a seven-point Likert scale (strongly disagree to strongly agree). A sample statement is “The leader is accessible for discussing emerging problems.” Cronbach’s alpha for IL in the current study is 0.86. *Work Engagement* was measured with a nine-item validated Chinese version (UWES-9) of the Utrecht Work Engagement Scale on a seven-point Likert scale (0 = never, 6 = always) recently used by Cheng et al. (2020) with a three-factor model (vigor, dedication, and absorption) consisting of three items each. A sample statement is “When I get up in the morning, I feel like going to work.” Cronbach’s alpha was 0.91. *Psychological Distress* was measured through the six-item K6 scale developed by Kessler and Mroczek (1994) for non-specific psychological distress over the past 30 days (0 = none to 4 = all the time) with a Chinese version validated by Jang et al. (2018). Cronbach’s alpha was 0.86. Coworkers’ self-sacrificial behavior was measured with a five-item *Self Sacrifice* scale adopted and modified from Mostafa and Bottomley (2020). A sample statement is “My coworkers are willing to make personal sacrifices in the team’s interest”. Cronbach’s alpha was 0.88.

### Common Method Bias and Marker Variable

Temporal separation was used to avoid common method bias as recommended by Podsakoff et al. (2003) and Cortina and Landis (2013) for studies using a cross-sectional design wherein responses have a possibility of being affected by respondents’ social desirability. The sequence of questionnaire items was changed for all constructs. These steps helped ensure common method bias did not contaminate the data. Computer self-efficacy (three items) was adopted from Taylor and Todd (1995) for use as an *a priori* measured marker variable (Chin et al., 2013). It was theoretically unrelated to the main model constructs. Data were collected for the marker variable before the data for the main constructs. The difference between the values of R-squared in work engagement and psychological distress before and after adding the marker variable was less than one percent, well below the suggested 10 percent limit (Chin et al., 2013). Additionally, full collinearity checks for variance inflation factor (VIF) values suggested by Kock (2015) and more recently used by Raza et al. (2020) were run, and inner VIF values were lower than three at the factor level, suggesting there was no common method bias. As recommended by Schaufeli (2004), in order to avoid response bias resulting from specific connotations related to the term “work engagement,” the words “work and well-being” were used in the questionnaire.

## Analysis and Results

The data were analyzed with SmartPLS 3.2.9 (Ringle et al., 2015) using partial least-squares structural equation modeling (PLS-SEM) which performs better for predictive models. Moreover, it simultaneously estimates relationships between multiple independent and dependent variables (of the structural models) and the latent, multiple observed or unobserved constructs of measurement model (Sarstedt et al., 2017). Recent advances in PLS-SEM research (Hair et al., 2019) show that it is a superior method for analyzing mediation relationships. Based on past studies on psychological distress (Eriksen et al., 2006; Stuke and Bermpohl, 2016), control variables were studied by splitting data into groups based on working hours, age, gender, education, and experience; then a bootstrap was run using multi-group analysis (MGA) in smartPLS to examine whether these factors caused differences in responses. However, these factors proved not to be significant and were thus removed from the structural model.

### Psychological Distress Scores

As recommended by Prochaska et al. (2012) in their study of a sample of 50,880 adults, optimal lower threshold cut-points for K6 were set as moderate ≥5 and severe ≥13. Scores of psychological distress (Mean = 5.28, Std. Deviation = 3.98) are given in Table 2, showing 65 percent of the respondents experienced moderate to severe psychological distress during the COVID-19 pandemic.

**TABLE 2:** Scores of psychological distress.

Psychological distress level		Frequency	Percent
Negligible	1–4	174	35.0
Moderate	5–12	245	49.3
Severe	13–19	78	15.7
	Total	497	100

### Work Engagement Scores

Based on the norms devised by Schaufeli (2004) in a study validating UWES with over 12,000 respondents across 10 countries, Table 3 compares the work engagement scores of Chinese nurses to conclude they displayed high levels of vigor and dedication and very high levels of absorption in their work during the COVID-19 pandemic.

**TABLE 3:** Level of work engagement.

	This study		Norms recommended by Schaufeli (2004)				
	Mean*	*SD*	Very low	Low	Average	High	Very high
Vigor	5.39	0.46	≤2.00	2.01–3.25	3.26–4.80	*4.81*–5.65*	≥5.66
Dedication	5.51	0.41	≤1.33	1.34–2.90	2.91–4.70	*4.71*–5.69*	≥5.70
Absorption	5.43	0.45	≤1.17	1.18–2.33	2.34–4.20	4.21–5.33	≥5.34*
Total	5.44	0.41	≤1.77	1.78–2.88	2.89–4.66	*4.67*–5.50*	≥5.51

*Values in bold italics represent category this study’s scores fall into; SD, Standard Deviation.

### Assessment Results for Measurement Model

Internal reliability was established through composite reliability (CR). All the constructs had CR scores higher than 0.80 indicating sufficient reliability (Table 4). The criterion to retain an indicator was set at a value of 0.50 for the item loading (Hair et al., 2017), which ranged between 0.502 and 0.943. One item for IL with a loading below 0.40 was removed (Hair et al., 2017). Convergent validity was confirmed through the Fornell and Larcker (1981) criterion as all average variance extracted (AVE) values were higher than 0.50. The discriminant validity (Table 5) was assessed through Fornell-Larcker as well as heterotrait-monotrait (HTMT) criteria developed by Henseler et al. (2014). HTMT values (top right of the diagonal values in bold) were below the conservative threshold of 0.85 while the Fornel-Larcker criterion was also fulfilled (Sarstedt et al., 2017).

**TABLE 4:** Reliability and convergent validity.

	Mean	*SD*	Composite reliability (CR)	Average variance extracted (AVE)
PD	5.28	3.98	0.920	0.704
IL	6.53	0.36	0.896	0.521
ESS	4.66	0.39	0.876	0.544
WE	5.44	0.41	0.945	0.852

**TABLE 5:** Discriminant validity.

Constructs	ESS	IL	PD	WE
ESS	0.839*	0.832	0.350	0.464
IL	0.736	0.721*	0.399	0.729
PD	−0.357	−0.480	0.738*	0.322
WE	0.425	0.654	−0.422	0.923*

*Bold italic values represent square root of AVE; HTMT values are above the diagonal.

### Assessment Results of Structural Model

A 5,000-sampled bias-corrected bootstrap was run with a 95 percentsignificance level to generate the *t*-values using a two-tailed estimation (Sarstedt et al., 2017). Based on the *t*-value rule of thumb for interpretation of a two-tailed test, i.e., 1.96, the hypothesized relationships proved significant (Table 6). Inclusive leadership has a negatively correlated relationship with psychological distress (β = −0.367, *t* = 3.205) and positively correlated with work engagement (β = 0.653, *t* = 27.174), explaining 42.6 percent of its variance. As for the mediating role of work engagement, the specific indirect effect was significant (β = −0.157, *t* = 4.64) and proved to be a complementary mediator because inclusive leadership also has a significant negative effect on distress. Together, inclusive leadership and work engagement explain the 33 percent variance in psychological distress.

**TABLE 6:** Structural model results.

Path	β	Mean	*SD*	*T*-value	*P*-Value	2.5% CI	97.5% CI	Hypotheses remarks
IL →PD	−0.367	−0.37	0.11	3.21	0.001	−0.611	−0.158	H1 = Supported
IL →WE → PD	−0.157	−0.16	0.03	4.64	0.001	−0.222	−0.089	H2 = Supported
Moderation-ESS	−0.269	−0.26	0.06	4.79	<0.001	−0.383	−0.165	H3 = Supported
IL → WE	0.653	0.66	0.02	27.17	<0.001	0.600	0.695	–
WE → PD	−0.240	−0.24	0.05	4.84	<0.001	−0.330	−0.139	–

*β, Path Coefficient; CI, Confidence Interval Bias Corrected; S.D., Standard Deviation.*

**FIGURE 2:**
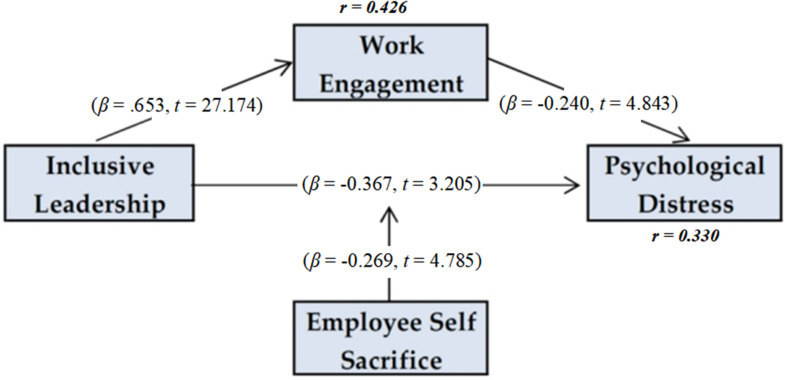
Results of the structural model.

**FIGURE 3:**
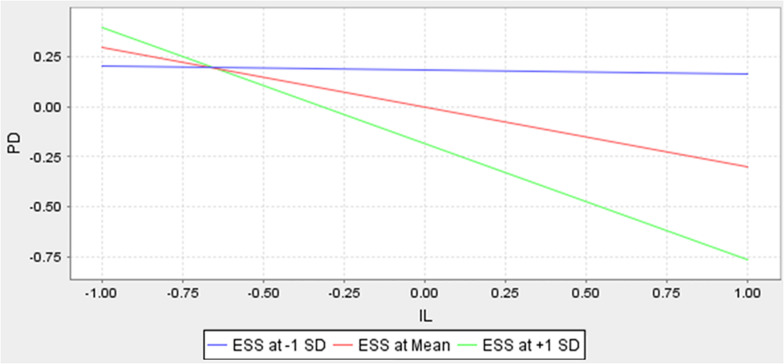
Moderation Effect.

Figure 2 depicts the results of the model with path coefficients, *t*-values are in parentheses and r square values are displayed within the constructs. The moderating role of ESS proved to be statistically significant (β = −0.269, *t* = 4.79). The relationship was plotted through smartPLS simple slope option to show effects at −1 standard deviation (SD) and +1 SD compared with a mean SD value of self-sacrifice. All relationships were significant at *p* < 0.001. Figure 3 depicts the moderating effect plotted in a graph with inclusive leadership along the *x*-axis and psychological distress along the *y*-axis.

The model strength and quality (Table 7) was assessed through benchmarks for effect size indicated by *f*-square values (>0.02) and predictive relevance indicated by Q square values (> 0) as suggested by Sarstedt et al. (2017). The effect size of all relationships proved in line with recommended benchmarks.

**TABLE 7:** Model strength and predictive relevance.

	f Square		Q Square		
	
	PD	WE	SSO	SSE	Q^2^ (=1 - SSE/SSO)
IL	0.039	0.74	3976	3976	
Moderating effect-ESS	0.05		497	497	
PD			2982	2761.043	0.074
WE	0.039		1491	957.258	0.358

## Discussion

This paper is intended to provide the results of the study into the relationship of inclusive leadership with psychological distress while evaluating the mediating mechanism of work engagement and moderating role of self-sacrifice. Previous research has focused on the management of nurses under normal circumstances (Choi et al., 2017; Masood and Afsar, 2017) rather than in the situation of an epidemic such as COVID-19. Therefore, this study compensates for this gap and confirms the results of past studies (Choi et al., 2015) while extending those results through empirically indicating the mediating role of work engagement. The results suggest inclusive leaders should work to create more open and engaging environment for nurses. This, in turn, helps improve employees’ vigor, focus, and engagement while reducing psychological distress.

The results affirm past research on the influence of positive leadership styles in reducing the psychological distress of respondents who experienced traumatic events (Birkeland et al., 2015). By contrast, the results of this study contradict some previous studies in which head nurses were found to exhibit very low levels of inclusive leadership (Wang et al., 2019), and studies in which the findings indicated positive leadership styles were not significantly related to long-term psychological distress (Nielsen et al., 2018). However, these past studies were conducted under normal circumstances and not during an actual public health emergency such as COVID-19. These results also differ from a study on PD and the well-being of nurses (Van der Heijden et al., 2017), which found that the social support from immediate supervisors was not a significant predictor of PD.

The current study’s findings indicate that Chinese nurses had a high level of work engagement, which contradicts past studies claiming lower work engagement among Chinese nurses due to high work pressures and imbalances between effort and reward (Kong and Li, 2018; Wang et al., 2019). The reason for this difference could be explained by the present circumstances of the pandemic. Empirical results of this study indicate the mediating role of work engagement between inclusive leadership and psychological distress, which is similar to the findings of previous studies indicating that a higher level of work engagement appears to reduce risks of onset of major depressive episode (Imamura et al., 2016), and work engagement appears to have positive relationship with inclusive leadership (Wang et al., 2019). Longitudinal studies have shown a positive association of work engagement with quality of life and productivity through increased staff engagement and commitment (Stinglhamber and Vandenberghe, 2003; Elovainio et al., 2013). However, these results also contradict past research by de Beer et al. (2014) whose cross-sectional study reported that work engagement had no significant relationship with any health conditions, such as depression or hypertension.

The results of the current study provide indirect support for the social exchange theory. When followers face a state of uncertainty during the COVID-19 pandemic, they tend to show keen interest in the supportive behavior and interaction of leaders as they seek to mitigate their followers’ fear and safety concerns. Nurses reciprocate the inclusive behavior of leaders and improve work engagement to return the support to those leaders who have helped them reduce stress and ensure their psychological well-being. Subordinates observe the leaders under uncertain circumstances. If a leader sacrifices his/her individual benefits for the collective welfare, followers are highly likely to attribute this behavior to a desire to serve the collective, thus catalyzing high work engagement among subordinates and reducing psychological distress. Inclusive leadership meets nurses’ socio-emotional needs for approval through supportive behavior, affiliation, and esteem. This leads to increased commitment (Pepe et al., 2019) and motivates employees to reciprocate by exhibiting enhanced work engagement to the organization (Kurtessis et al., 2017).

The current study’s empirical evidence suggests the moderating role of self-sacrificial behavior in enhancing the effect of inclusive leadership on psychological distress. The social exchange theory not only explains the exchange relationships of reciprocity between leaders and followers, but also the ethical conduct at work and moral indebtedness, which creates a self-sacrificial inclination within coworker relationships. Self-sacrifice is a fast and more intuitive response (Belanger et al., 2014). During the COVID-19 outbreak, nurses have sacrificed their daily self-interests for the collective need of their peers in an environment inspired by support through inclusive leadership.

## Practical Implications

This study has implications with respect to implementation of comprehensive guidelines by the Chinese National Health Commission (NHC, 2020) issued on January 27, 2020, for psychological crisis intervention during the COVID-19 outbreak. These guidelines emphasize extending mental health support to health care workers through multi-disciplinary teams. Emphasizing an inclusive and support behavior among members of nursing and health care leadership teams can be an appropriate strategy as it can operationalize the real-time delivery of support initiatives during an epidemic in work group settings at the hospitals.

In their invited commentary on the task-force initiative in New York City for health care workers during the COVID-19 pandemic, Ripp et al. (2020) reported that paying attention to the “emotional well-being of health care workers has emerged as a central element in the COVID-19 response” (p. 5). For this purpose, one of the three priority areas included providing enhanced communication for delivering reliable and reassuring messages. The practice of inclusive leadership may also serve to preclude the need for psychiatric services, which possibly mitigates as an alternative to psychological counseling for nurses suffering from psychological distress during their duties. It also saves them from the fear of stigma if they lessen their work engagement to seek help from a psychiatrist. Research has shown that the stigma of being infected by a pathogen and the stigma of being affected by depression are an inseparable reality in the nursing profession (Nyakarahuka et al., 2017; Brooks et al., 2020; Petzold et al., 2020).

These guidelines emphasize extending mental health support to health care workers through multi-disciplinary teams. In addition, cognitive behavior therapy and mindfulness-based therapy are helpful during the COVID-19 pandemic (Ho et al., 2020). The concept of social exchange is not limited to support from leaders; it also includes peer support through unselfish and self-sacrificial behavior. These behaviors can be interpreted through small acts of kindness and favors between colleagues. For example, younger nurses switching shifts with older nurses to help them get quality sleep; identifying stressed behavior and providing a listening ear; or taking up longer work hours to help colleagues with children or older parents all demonstrate selfless behavior. Inclusive leadership can help protect the mental health of nurses if nursing managers act to ensure that their subordinates are supportive of their coworkers during an epidemic. This tends to generate an environment of self-sacrificial behavior for each other in the long run and enhances the effects of the inclusive leadership style in reducing occurrences of psychological distress.

During an outbreak such as the COVID-19 pandemic, nurses are surrounded by disease, pain, suffering, and death. They witness patients suffering and dying of an infectious disease, and it makes them feel hopeless and even worthless. In China, head nurses are primary administrators responsible for managing clinical nursing as well as matters related to patient care and hospital operations while coordinating between doctors, patients, and nurses (Wang et al., 2019). Positive leadership styles such as inclusive leadership help subordinates stay mentally strong to continue fighting the infectious disease. When nurses feel they are being cared for by leaders using inclusive behavior, positive psychological conditions such as feelings of safety and meaningfulness are the result.

With respect to the implications of the premise of the theory of shattered assumptions by Janoff-Bulman (1992), epidemic situations such as those caused by the Severe Acute Respiratory Syndrome Coronavirus-2 (SARS CoV-2) bring trauma and shatter employees’ perceptions of the world as a safe place. Inclusive leadership serves to comfort and assure workers in the midst of volatile, uncertain, complex, and ambiguous situations. Employees tend to appreciate a leader who is open and available and recognizes the employees’ anxiety caused by perceived or actual threats. The leader’s attitude and behaviors tend to be contagious and serve to reduce stress among employees (Goleman et al., 2013; Tucker et al., 2020). Therefore, leaders should focus on providing, as much as practical, a psychologically and physiologically safe environment for nurses in their respective hospitals.

Improving nurses’ engagement during an outbreak helps inclusive leaders mitigate psychological distress among nurses while inculcating a culture of openness, availability and accessibility. Inclusive leadership creates an environment that facilitates meaningfulness and a sense of safety in which nurses find strength through support of leaders. Personnel who are not in formal leadership roles should be trained to create inclusive environments in order to provide effective depth in leadership and will serve to also help inspire work engagement among subordinates. A mentally healthy and psychologically relaxed nurse is less likely to make mistakes, thus reducing adverse events within hospitals while also improving patient outcomes. Leadership training in supportive behaviors should be made part of the induction programs, with incentive programs for inclusive leadership to create a collaborative environment with low power distance between subordinates and leaders.

## Limitations and Future Research

This study discusses nursing staff only, and caution is advised with respect to generalizing the findings to another cadre, industry, or type of firm. Cross-sectional design was a limitation of this study with single-source data, although there was no contamination due to common method bias. For future studies, it is suggested that multi-source studies on inclusive leadership in health care be conducted with additional outcome variables related to extra-role behaviors of employees reported by peers or supervisors. This study was conducted in Chinese hospitals in Wuhan at the epicenter of the COVID-19 outbreak. Its generalizability may be improved through further research by drawing samples from American, Australian, or European regions currently suffering from a highly infectious phase of COVID-19, and wherein nurses are highly likely to face psychological distress. Another direction for future research is a longitudinal study on inclusive leadership comparing inclusive leadership’s effects on psychological distress during an epidemic with its effects under normal circumstances after the pandemic is over. A similar study was recently conducted by Wang et al. (2020) for observing psychological distress levels among the general public. This type of study will contribute to establishing whether the impact of inclusive leadership and work engagement on psychological distress varies in the long run after the pandemic is over, by means of extending a research effort into a longitudinal study within work settings. The final limitation of this study did not assess leadership related to COVID-19 to ensure a safe environment for nurses that was found to protect mental health in the general workforce in a recent study by Tan et al. (2020).

## Data Availability Statement

The dataset collected for this study can be found in the Mendeley Data repository: (https://data.mendeley.com/datasets/4vg8xtsw5t/1).

## Ethics Statement

Ethical review and approval was not required for the study on human participants in accordance with the local legislation and institutional requirements. Written informed consent to participate in this study was provided by the participants.

## Author Contributions

FA and FZ: conceptualization, funding acquisition, and project administration. FA and NF: data curation. NF: formal analysis and software. FA: investigation and writing – original draft. FA, FZ, and NF: methodology. All authors contributed to the article and approved the submitted version.

## Conflict of Interest

The authors declare that the research was conducted in the absence of any commercial or financial relationships that could be construed as a potential conflict of interest.

## Footnotes

1 wenjuan.com
